# Nano-scale microfluidics to study 3D chemotaxis at the single cell level

**DOI:** 10.1371/journal.pone.0198330

**Published:** 2018-06-07

**Authors:** Corina Frick, Philip Dettinger, Jörg Renkawitz, Annaïse Jauch, Christoph T. Berger, Mike Recher, Timm Schroeder, Matthias Mehling

**Affiliations:** 1 Department of Biomedicine, Basel University and University Hospital Basel, Basel, Switzerland; 2 Department of Biosystems Science and Engineering, ETH Zürich, Basel, Switzerland; 3 Institute of Science and Technology Austria (IST Austria), Klosterneuburg, Austria; 4 Department of Neurology Department, University Hospital Basel, Basel, Switzerland; Katholieke Universiteit Leuven Rega Institute for Medical Research, BELGIUM

## Abstract

Directed migration of cells relies on their ability to sense directional guidance cues and to interact with pericellular structures in order to transduce contractile cytoskeletal- into mechanical forces. These biomechanical processes depend highly on microenvironmental factors such as exposure to 2D surfaces or 3D matrices. *In vivo*, the majority of cells are exposed to 3D environments. Data on 3D cell migration are mostly derived from intravital microscopy or collagen-based *in vitro* assays. Both approaches offer only limited controllability of experimental conditions. Here, we developed an automated microfluidic system that allows positioning of cells in 3D microenvironments containing highly controlled diffusion-based chemokine gradients. Tracking migration in such gradients was feasible in real time at the single cell level. Moreover, the setup allowed on-chip immunocytochemistry and thus linking of functional with phenotypical properties in individual cells. Spatially defined retrieval of cells from the device allows down-stream off-chip analysis. Using dendritic cells as a model, our setup specifically allowed us for the first time to quantitate key migration characteristics of cells exposed to identical gradients of the chemokine CCL19 yet placed on 2D vs in 3D environments. Migration properties between 2D and 3D migration were distinct. Morphological features of cells migrating in an *in vitro* 3D environment were similar to those of cells migrating in animal tissues, but different from cells migrating on a surface. Our system thus offers a highly controllable *in vitro*-mimic of a 3D environment that cells traffic *in vivo*.

## Introduction

Directed cell-migration is fundamental to many physiological processes including embryogenesis, wound healing and, importantly, also in immunity [1]. Orchestrated migration of immune cells provides the basis for their precise positioning within lymphoid and non-lymphoid tissues which is essential for pathogen defense and cancer immunosurveillance [1]. On the other hand, altered migration of immune cells is fundamental to the pathogenesis of various diseases such as autoimmune diseases or primary immunodeficiencies [2, 3]. Migration of immune cells is guided by extracellular directional guidance cues such as chemokine gradients [4] and relies on the ability of cells to interact with extracellular substrates [5]. Specifically, binding of integrins to extracellular matrix molecules allows the formation of focal adhesion sites that link the actin cytoskeleton with the substrate surrounding the cell [6]. This interaction provides the basis for transducing contractile forces of the cytoskeleton to mechanical forces that are required for locomotion [7]. Alternatively, in 3D environments dendritic cells (DCs) can also migrate in the absence of integrins by squeezing of the cell body through the pericellular environment [8]. Under physiological conditions only 2–5% of immune cells circulate in blood and are therefore exposed to the 2D surfaces of luminal blood vessel endothelial cell surfaces [9]. On the contrary, the vast majority of immune cells are positioned in lymphoid and non-lymphoid tissues and are accordingly exposed to 3D tissue environments [9]. Therefore, *in vitro* migration assays that allow tracking of cells moving on 2D surfaces do not provide microenvironmental conditions immune cells are mainly exposed to. As a result, the majority of our functional understanding of integrin-dependent and integrin-independent 3D cell migration is based on findings of 2-photon microscopy in living animals. These *in vivo* approaches are expensive and the surgical preparation of the animals inherently induces local inflammation and an alteration of soluble factors. This limits the potential to assess the influence of microenvironmental effects such as cytokine niches or chemokine gradients on immune cell migration [10]. As a consequence, *in vitro* assays have been developed that allow assessment of 3D immune cell migration in diffusion-based chemokine gradients [11]. Cells migrating in such 3D migration assays share key phenotypical features with cells that migrate *in vivo* [12]. However, these *in vitro* assays do not allow to precisely control defined microenvironmental factors such as chemokine gradients. Also, harvesting of cells according to their potential to migrate in 3D environments is not possible. Therefore, fundamental aspects of immune cell migration are difficult to assess mechanistically by these *in vivo* and *in vitro* experimental approaches.

To address these limitations, we developed an automated microfluidic system that allows positioning of immune cells in 3D microenvironments containing fully controllable diffusion-based chemokine gradients, tracking of cells in real time at the single cell level [13] and spatial deterministic retrieval of cells from the device. Using DCs as a model our setup allowed us for the first time to quantitatively assess migration on 2D vs in 3D environments in similar gradients of the chemokine (C-C motif) ligand19 (CCL19) and compare morphological properties in these conditions with that of cells migrating in animal tissues.

## Material and methods

### Mice

C57BL/6 mice used for *in vitro* migration assays were bred and maintained under pathogen-free conditions at the University of Basel, Department of Biomedicine animal facility. Mice with an age of 8–12 weeks were used for the study. All experimental procedures were in strict accordance with the veterinary office regulations of the Canton of Basel-Stadt, Switzerland (authorisation nos. 2772 and 2875).

C57BL/6J mice used for in ear migration assays were bred and maintained in accordance with the Austrian law for animal experiments (“Österreichisches Tierschutzgesetz”) and sacrificed at 4 to 10 weeks of age for use in experiments. Permission and all experimental protocols were approved by the Austrian Federal ministry of science, research and economy (identification code: BMWF-66.018/0005-II/3b/2012).

### Cell isolation, culture and dendritic cell maturation

Dendritic cells (DCs) were generated from bone marrow cells extracted from femur and tibia of mice. In brief, bone marrow cells were collected by flushing the bones with PBS. Next, 2.5x10^6^ bone marrow cells were cultured in 10 mL R10 cell culture medium (RPMI 1640 (Sigma-Aldrich) containing 10% fetal calf serum (FCS), 1% Penicillin, 1% Streptomycin and 50 μM 2-Mercaptoethanol, all from Invitrogen) containing 20 ng/mL granulocyte-macrophage colony-stimulating factor (GM-CSF) derived from the supernatant of a hybridoma cell line in a non-adhesive petri dish. On day 3, 10 mL of R10 medium containing 20 ng/mL GM-CSF were added. On day 6, 10 mL of cell culture medium was replaced by 10 mL of R10 medium containing 20ng/mL GM-CSF. DCs were cryopreserved on day 8 of the culture. Thawed DCs were maturated overnight with 200 ng/mL lipopolysaccharide (LPS, Sigma-Aldrich) and 20 ng/mL GM-CSF derived from supernatant of a hybridoma cell line in an adhesive cell culture dish.

B cells were isolated by positive selection (CD19 MicroBeads, Miltenyi) from mouse splenocytes followed by positive selection (CD3ε MicroBeads, Miltenyi) of T cells from the run-through. Cell purity assessed by flow cytometry was 92%.

### Design and fabrication of microfluidic chips

Microfluidic photomasks were designed and silicon wafers fabricated as described earlier [14]. Also, microfluidic chips were fabricated by multi-layer polydimethylsiloxane (PDMS) soft-lithography as described previously [15]. Briefly, we drafted a two layer design using AutoCAD (Autodesk Inc., San Rafael, CA, USA) which was then printed on transparencies at 40kdpi resolution (Fine Line Imaging, Minneapolis, USA) and replicated on 4” silicon wafers using standard photolithography procedures [16] for SU-8 3025 (Michrochem, Westborough, MA, USA) and parabolic AZ-50XT (AZ Electronic Materials, Luxembourg). Flow layer wafers were then covered with PDMS (10:1 polymer: catalyst) and debubbled before curing at 80°C for 45 min. Control layer wafers were subsequently spin-coated with PDMS (20:1 polmer: catalyst) at 1800 rpm before curing at 80°C for 45 min. Flow layer inlets were punched in the cured flow layer before plasma treatment and alignment to the control layer. Finally, control layer inlets were punched and the assembled chips were plasma treated before glass bonding and a 16 h incubation at 80°C to complete the bonding process.

### Chip set-up, operation and control

The glass slide carrying the microfluidic chip was cleaned and taped on a slide holder. Control channels were connected to miniature pneumatic solenoid valves (Pneumadyne) that were controlled via an established control box system [17] with a custom Matlab (MathWorks) graphical user interface. Optimal closing pressures of push-up PDMS membrane valves were determined individually for each chip. Following this, the pressure in control channels was increased by 0.2 bar. Flow lines were connected to inlets, pressurized with 0.1 bar and the whole chip except the migration chambers was filled with phosphate buffered saline (PBS). Flow channels and waste outlets were treated with pluronic F-127 (10 μg/mL, Invitrogen) for 3 min while the migration chamber valves remained closed to prevent exposure to pluronic. This was followed by extensive washing with PBS for 20 min. Following incubation of cell culture chambers for 1 hour with human plasma fibronectin (100 μg/mL, Millipore), the entire chip was flushed with cell culture medium.

### Generation and visualization of a microfluidic 3D collagen matrix

Bovine Collagen I solution (PureCol, Advanced BioMatrix) was neutralized with 0.4% sodium bicarbonate and 10x minimum essential medium eagle (MEM) (all from Thermo Fisher Scientific) prior to mixing at a volume ratio of 1:2 with RPMI cell culture medium containing mature DCs at a concentration of 30 x 10^6^ cells per mL resulting in a final collagen concentration of 1.7 mg/mL and cell concentration of 10 x 10^6^/mL. For final collagen concentration of 0.8 mg/mL the bovine collagen solution was pre-diluted with PBS 1:2. For a final collagen concentration of 3.2 mg/mL a more concentrated bovine collagen I solution (Nutragen, Advanced BioMatrix) was used. The gel containing mature DCs was immediately loaded into microfluidic migration chambers. Following this, the valves connecting the microfluidic migration chambers with the flow lines were closed and flow lines were washed extensively with PBS to prevent unwanted clotting of collagen fibers in flow channels. Collagen was allowed to polymerize for 60 minutes inside the migration chambers. Staining of polymerized collagen was performed by gently flushing a rabbit monoclonal anti-collagen I antibody (EPR7785, abcam) diluted 1:100 in PBS containing 2% FCS across the migration chambers. Following incubation for 30 min and washing, an AF647 donkey anti-rabbit IgG (Invitrogen) diluted 1:100 in PBS containing 2% FCS was gently flushed across the migration chambers, incubated for 30 min and washed with PBS. In order to dynamically visualize the collagen polymerization both antibodies were directly added to the collagen monomer suspension, loaded on the chip and imaging was performed every 2 minutes for 1 hour. Collagen staining was quantified following background subtraction and averaged across the same area using the image analysis software Fiji.

### Generation of soluble chemokine gradients

Diffusion-based chemokine gradients were generated and maintained as previously described by using a switching source-sink flow pattern [17]. Briefly, the source channel at the upper short end of the cell culture chamber was filled with fresh cell culture medium (R10) containing murine CCL19 (5 μg/mL, PreproTech) and FITC-dextran 10 kDa (100 μg/mL, Sigma-Aldrich) or AF647-dextran 10kDa (100 μg/mL, Invitrogen). Then the flow was stopped and the valve connecting the source channel with the chamber was opened for 5 sec while the valve connecting the sink channel with the chamber at the opposite end was kept closed. The same procedure was repeated at the sink channel with cell culture medium. By doing so, a local high concentration (source) and a low concentration (sink) is established between which a chemokine gradient is built up within 60 min and maintained by diffusion. The fluorescently labelled-dextran gradient served as a proxy to monitor the chemokine gradient within the chamber and was imaged every 10 min.

### Imaging, cell-tracking and data analysis

Cells and collagen gel polymerization were imaged using an automated inverted microscope (Nikon Ti, 10×/NA 0.45 Air Plan Apo OFN25 DIC N1, 20x/NA 0.45 Air Plan Fluor ELWD or 40x/NA 0.6 Air Plan Fluor ELWD objective; Nikon) equipped with a custom-made stage-top incubator with temperature control connected to a gas incubation system (ibidi, Germany; settings: CO_2_-concentration: 5%, humidity: 80%), a digital CCD camera (CCD C10600-10B; Hamamatsu photonics) or a digital CMOS camera (C13440-20CU, Hamamatsu photonics) and the imaging software Nikon NIS-AR (Nikon). For quantification of migration properties, cells were imaged every 20 seconds for 90–120 min. Cell tracks are represented on an x-y coordinate system, with the origin of each trajectory aligned to (0,0). Each track is color-coded for time, such that yellow colors represent early and red colors later time points. For image processing and cell tracking, the analysis software Fiji and a plugin for manual tracking (“Manual Tracking”, Cordelieres 2005) were used. Tracking data were analyzed using a custom analysis algorithm programmed in R. The following readouts of cell migration were calculated: velocity (calculated by distance of the migration track divided by time, [μm/min] and Chemotactic Index as a measure of directedness of cell migration towards the gradient (calculated by dividing the distance from start to end point of cells in axis of the gradient (Y-Displacement) by the total migration distance of every cell). Morphological characteristics of cells migrating on 2D vs in 3D environments were quantified by the elongation factor (longest diameter of an individual cell divided by its perpendicular width). Elongation factor was measured at two time points of the time-lapse and pooled. Rose plot summarize directions of every cell movement relative to the starting point. Each section covers an angle of 11.25°. Where indicated DCs were labelled with CellTracker Deep Red dye (100nM, Invitrogen).

### Immunocytochemistry of cells in microfluidic migration chamber and quantification

Following spontaneous or CCL19-directed migration in a collagen matrix cells were incubated for 5 min with R10 medium containing 1:100 diluted anti-mouse CD16/32 antibody (clone 93, biolegend) in order to prevent non-specific binding of immunoglobulins to the Fc receptors prior to incubation for 20 min with AF647 anti-mouse I-A/I-E antibody (clone M5/114.15.2, biolegend) diluted 1:100 in R10 medium containing anti-mouse CD16/32 antibody. Cells were washed with R10 medium by flushing across the migration chamber. Quantification of the staining intensity was performed in Fiji. Cell outlines were drawn on the bright-field image and then applied to the fluorescent image. Background subtraction was performed locally for every cell. Corrected total cell fluorescence (CTCF) was calculated as follows: Integrated Density–Area of selected cell * mean fluorescence of background readings).

### Cell harvesting from 3D collagen matrix

Following nuclear staining with DRAQ5 (biolegend), cells were loaded in a collagen suspension into microfluidic migration chambers and collagen was allowed to polymerize. After digestion of collagen with Liberase TL (Roche), cells were flushed via side channels to harvesting ports and transferred to micro-wells for imaging.

### Mouse ear crawl-in migration assay

Mouse ear sheets were prepared as previously described [18]. Briefly, following preparation of the dermis, ear sheets were mounted in custom-build chambers and fluorescently labelled (10 μM TAMRA, Invitrogen) mature DCs were added in phenol red-free R10 supplemented with 10 mM HEPES (Life Technologies) for live cell confocal microscopy imaging by using a confocal microscope (LSM700, Zeiss) equipped with an incubation system controlling for gas and temperature (37°C, 5% CO_2_).

### Statistical analysis

Two or more experiments were performed for each data set and one representative experiment is shown. Statistical analysis was performed in GraphPad Prim (Version 7.0c). Groups were compared using the non-parametric Kruksal-Wallis test with Dunn’s multiple comparison test. Not significant (ns) ρ ≥ 0.05; * ρ < 0.05; ** ρ < 0.01; *** ρ < 0.001; **** ρ < 0.0001. Plots were generated in R studio (Version 0.99.902) and boxplots show median, interquartile ranges (IQR) and whiskers indicate the 1.5x IQR or minimum/maximum values respectively.

## Results

### Microfluidic system to probe chemotaxis at the single cell level in 3D environments

**Fig 1:**
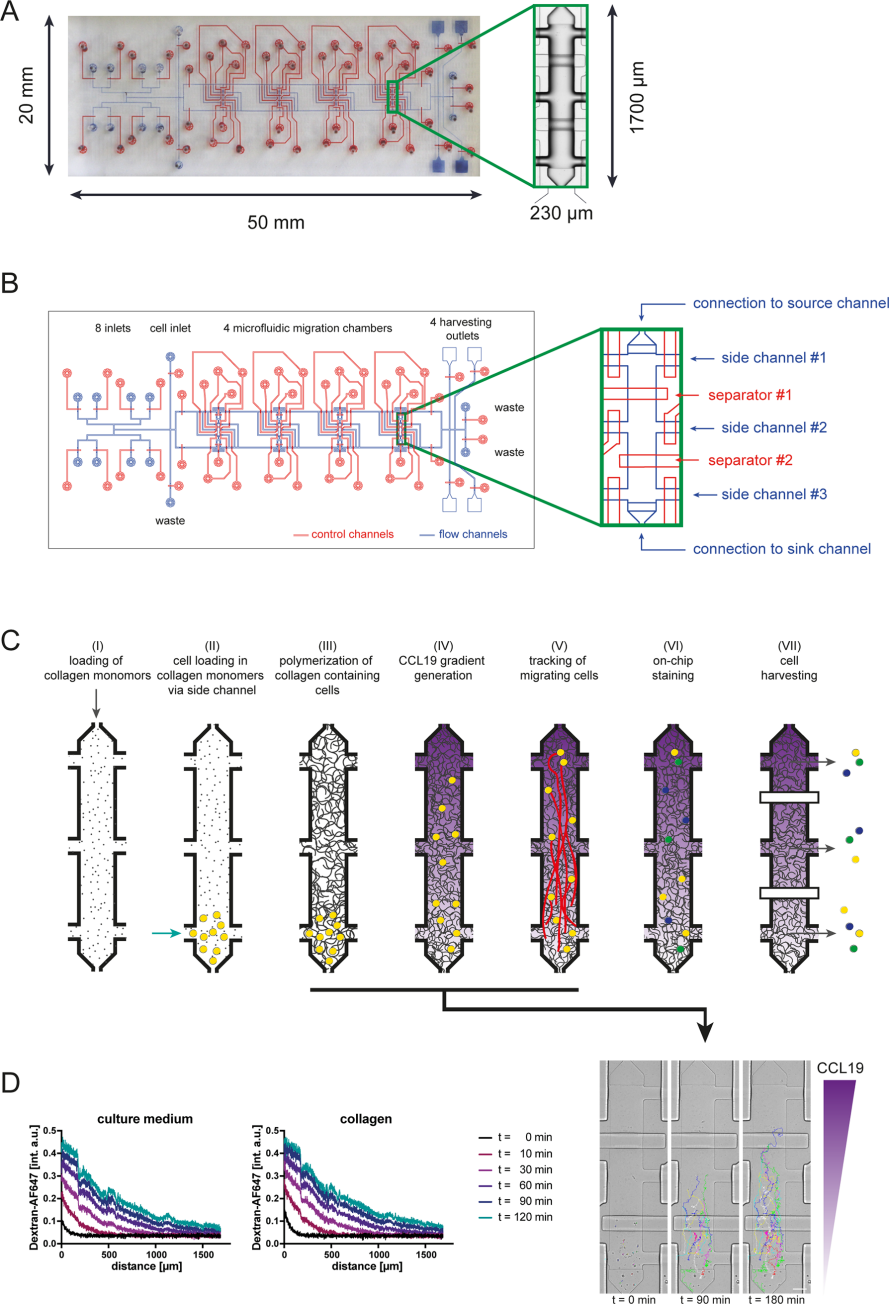
Geometry and functionality of collagen migration chip. (A) Photograph of the device, control channels are stained in red, flow channels are depicted in blue color. Inset shows air-filled migration chamber. (B) Schematic overview of device, inset shows migration chamber in detail. (C) Schematic overview of the functionality and workflow of the migration device. Insets show localized loading in migration chamber and tracking of dendritic cells migrating along a gradient of CCL19. Scale bar, 100 μm. (D) Comparison of gradient generation across migration chamber coated with fibronectin (100 μg/mL) and filled with cell culture medium or filled with collagen matrix (1.7 mg/mL). Gradient is visualized with a 10 kDa AF647-Dextran.

For assessing migration of cells in 3D environments we developed a microfluidic device that allows the generation of a collagen gel containing cells in microfluidic migration chambers. Specifically, the two-layer PDMS microfluidic device (overview in **Fig 1A**) consists of 8 inlets for reagents and media, 1 cell loading inlet, 3 waste outlets and 4 migration chambers (**Fig 1B**). The core component of this device are 4 migration chambers (l = 1700 μm, w = 230 μm, h_max_ = 28 μm, v = 7.4 nL) containing 3 individually controllable side ports at each of the 2 long ends of the chamber while the ports at the two short ends of the chamber are connected to supporting sink and source channels (**Fig 1B**). For controlled flow of fluids and cell suspensions all ports are equipped with independently controllable PDMS membrane valves. Support channels connect the reagent inlets with the migration chambers and the outlets. Cells can be loaded via the ports at the short ends resulting in a distribution along the chamber. Alternatively, cells can be loaded via 2 opposing ports at the long ends resulting in a localized distribution within the chamber, e.g. as schematically shown in **Fig 1C**. The implementation of 2 separating valves across the chamber allows physical partitioning of the chamber into 3 compartments. The functionality of the chamber with localized loading of cells is illustrated in **Fig 1C**. In detail, the design of the chamber allows (I) loading of collagen monomers, (II) cell loading in collagen monomers via side channel, (III) polymerization of collagen gel containing cells in localized area of microfluidic chamber, (IV) the generation of diffusion-based flow-free chemokine gradients in microfluidic migration chambers containing collagen gel, (V) tracking of cells migrating along chemokine gradients with time-lapse microscopy, (VI) phenotyping of cells following 3D migration by immunocytochemistry and (VII) retrieving of cells according to their position within the migration chambers for further off-chip analysis.

### Generation of 3D collagen gels in microfluidic migration chambers

In order to equip microfluidic migration chambers with a collagen gel, the pH of a cooled (4°C) acidic collagen monomer solution (pH = 2) was neutralized by the addition of minimal essential medium (MEM) and sodium bicarbonate. Next, cells were added and the collagen-cell-suspension was loaded into the microfluidic migration chamber. Exposure of the collagen-cell-suspension to cell culture conditions (37°C) and neutralization of the pH induced polymerization of the collagen monomers into collagen fibres within minutes **(S1A Fig, S1 Movie)**. The density of the collagen gel can be controlled by adjusting the concentration of the initial collagen monomer solution **(S1B and S1C Fig)**.

We have previously shown that the use of microfluidics allows the generation of flow-free diffusion-based chemokine gradients in which the steepness, mean concentration and duration can be independently controlled [14]. We therefore assessed next, whether the formation of a diffusion-based gradient is comparable between microfluidic chambers containing a collagen gel or cell culture medium only. To assess diffusion characteristics of chemokines under these conditions the built-up of gradients of fluorescent dextrans with a molecular weight that is similar to most chemokines (10 kDa) was quantitated. **Fig 1D** illustrates the dynamics of gradient generation in microfluidic migration chambers filled with cell culture medium or collagen gel. In both conditions, the gradient increases rapidly during the first 60 min followed by a reduced increment during the next 60 min. No differences were noted between the gradients generated in chambers containing collagen gel compared to chambers filled with cell culture medium only. Taken together, our setup allows the generation of similar diffusion-based chemokine gradients in 2D and 3D environments.

### CCL19-directed 3D chemotaxis, immunophenotyping and harvesting of cells

**Fig 2:**
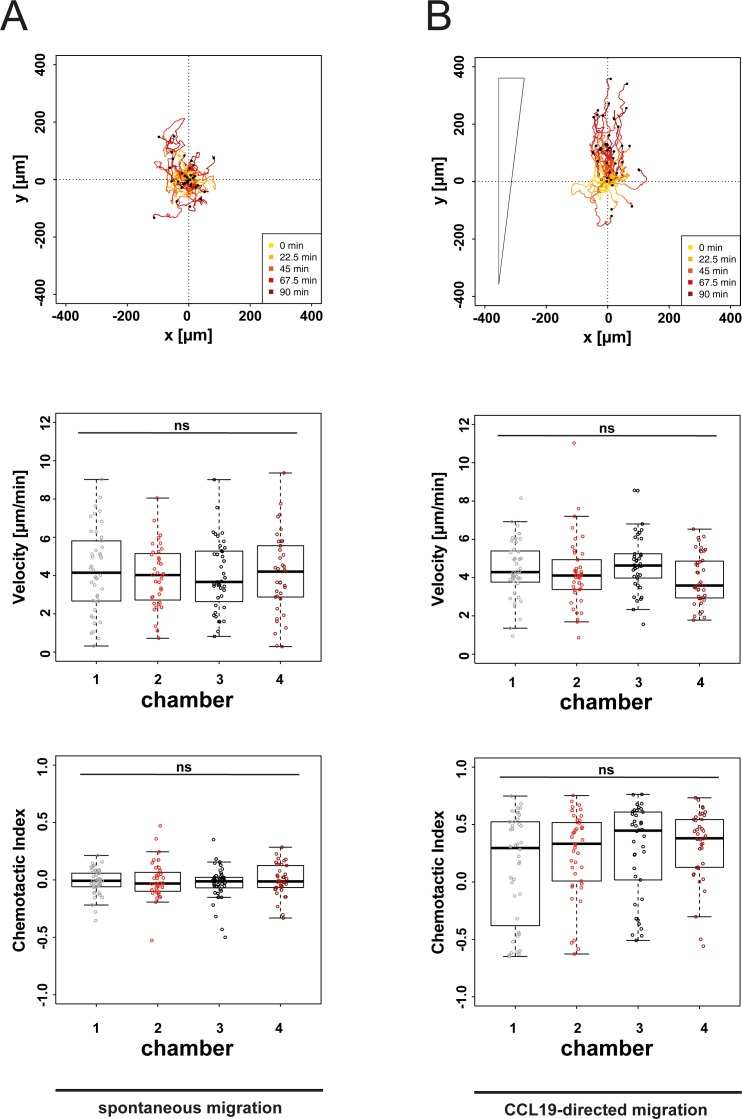
Reproducibility of microfluidic 3D migration assay. (A) Analysis of spontaneous migration of DCs in 4 distinct collagen preparations (collagen concentration: 1.7 mg/mL). Cells were tracked for 90 min during culture in cell culture medium only. Panel 1 exemplarily shows migration tracks plotted to a common starting point, migration characteristics are shown in panel 2–3. Chemotactic Index is a measure for chemotactic efficiency (calculated by dividing the distance from start to end point of cells in axis of the gradient by the total migration distance of every cell). (B) DCs were tracked for 90 min during diffusion-based soluble CCL19 gradient (maximal CCL19 concentrations: 5 μg/ml). Panel 1 exemplarily shows migration tracks plotted to a common starting point, migration characteristics are shown in panel 2/3. Median and interquartile ranges shown, ρ values were calculated by Kruksal-Wallis test with Dunn’s multiple comparison test. Not significant (ns) ρ ≥ 0.05.

We have previously shown that CCL19-directed chemotaxis can be induced in DCs in a microfluidic 2D environment coated with fibronectin [15]. To assess CCL19-directed migration of DCs in a 3D environment we polymerized within our microfluidic device a collagen monomer solution containing mature DCs. Following polymerization of the collagen gel, spontaneous migration of cells within the collagen gel was tracked by time-lapse microscopy for 90 min. DCs migrated under these control conditions spontaneously and undirected (**Fig 2A**, panel 1). To ensure reproducibility of our 3D system we replicated the quantification of spontaneously migrating DCs in 4 individual collagen preparations with the same cell suspension in all 4 chambers of our device in parallel. This revealed comparable mean velocities and levels of directedness (**Fig 2A**, panels 2 & 3). Exposure of DCs to gradients of the chemokine CCL19 induced in microfluidic 3D environments migration towards higher concentrations of the chemokine (**Fig 2B**, panel 1). To also ensure reproducibility of this 3D chemotaxis setup, DCs were exposed to CCL19 gradients along the long ends of all 4 chambers of our device in parallel. By doing so we observed in all 4 chambers comparable velocities of migrating cells (**Fig 2B**, panel 2). Also, the directedness of cell migration towards higher concentrations of the chemokine was comparable among the 4 individual chambers of our device (**Fig 2B**, panel 3). Since the gradient steepness varies across the migration chamber as shown in **Fig 1D**, we performed a sub-analysis comparing the cells migrating in the middle compartment of chambers. Cells initially located in the middle compartment of migration chambers showed a similar distribution of migration tracks, velocity and comparable chemotactic index values across chambers **(S2 Fig)**. These findings reflect the robustness of our setup to reproducibly quantify spontaneous migration and chemotaxis of primary cells under highly controllable conditions.

**Fig 3:**
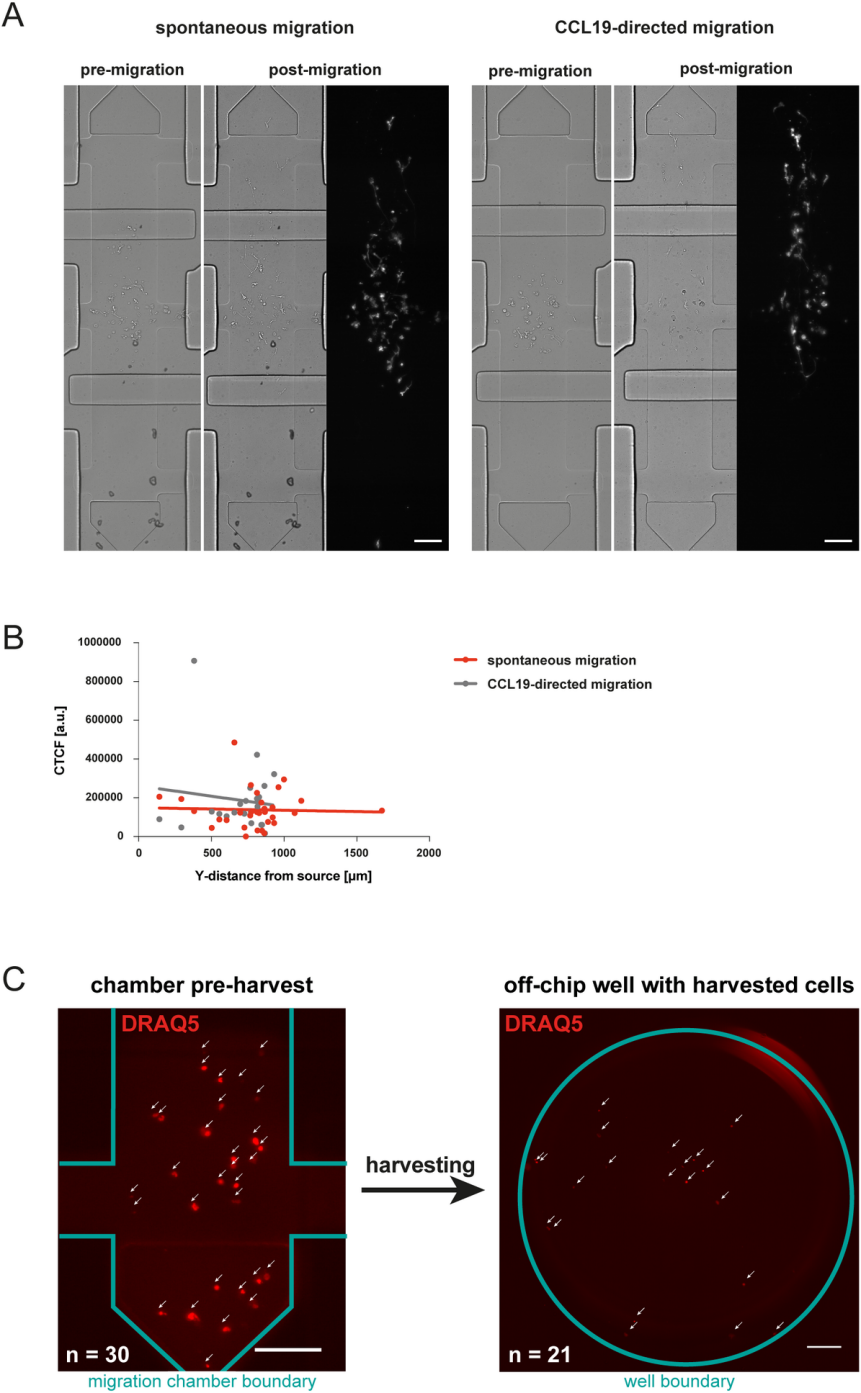
On-chip phenotyping and harvesting of cells. (A) DCs were loaded localized in the middle compartment of the migration chamber in a collagen gel (1.7 mg/mL) and allowed to migrate for 2h in cell culture medium or in a soluble CCL19 gradient (5 μg/mL). Cells were stained on-chip with an anti-mouse I-A/I-E antibody. Scale bar, 100 μm. (B) Quantification of anti-I-A/I-E (MHC class II) staining of cells. Corrected Total Cell Fluorescence (CTCF) (= Integrated Density–Area of selected cell * mean fluorescence of background readings) is shown relative to Y-position in migration chamber where 0 μm indicates the chemokine source and 1700 μm the sink of the gradient system. (C) Nuclear staining of DCs with DRAQ5, cells are shown in the microfluidic chamber prior harvesting (left panel, scale bar: 100 μm) and in micro well following harvest (right panel, scale bar: 200 μm).

In previous reports migration characteristics of immune cells in chemokine-gradients have been linked to the expression levels of molecules involved in chemotaxis [19, 20]. However, standard migration assays such as the transwell system do not allow to relate phenotypic with functional characteristics at the single cell level. To illustrate the capability of our setup to address this unmet need we loaded DCs locally in the middle compartment of the fibronectin-coated migration chamber and polymerized the collagen gel. After 2h of migration in cell culture medium or in a soluble CCL19 gradient we stained the cells with an anti-mouse I-A/I-E antibody on-chip (**Fig 3A**). This allows the quantification of the MCH-II expression in relation to the migratory and chemotactic capabilities of the cells as shown in **Fig 3B**. In DCs MHC-II expression has been linked to the maturation status [21] which conversely correlates with the migratory properties of DCs [22]. This prompted us to test in our setup the possibility that MHC-II expression might correlate with migratory properties of DCs. In matured DCs, expression levels of MHC-II varied considerably. However, MHC-II expression levels of individual cells did not correlate with migration properties towards a CCL19-gradient (**Fig 3B**). To test the specificity of the anti-MHC-II antibody used in our study, we assessed expression of MHC-II in B cells and T cells by on-chip immunocytochemistry. While B cells stained positive for MHC-II, no detectable expression of MHC-II was found on T cells **(S5 Fig)**. These findings are in line with previous reports on the expression of MHC-II in murine B cells and T cells [23, 24]. For additional off-chip analysis cells can subsequently be harvested in relation to their position in the chamber. To this end, the migration chambers were partitioned by the 2 separating valves into 3 compartments as indicated schematically in **Fig 1C** (panel VII). Next, the individual sections of the chamber were incubated with liberase to enzymatically degrade the collagen fibres. Following this, the contents of the respective chamber sections were flushed sequentially via the side-channels of the respective chamber section to different harvesting outlets (**Fig 3C**). Quantification of cells derived from the harvesting outlets revealed that consistently 70%–90% of the cells of a specific chamber region can be retrieved from the device. Taken together, this allows spatially deterministic harvesting of cells from the device, i.e. enrichment of cells sharing similar migration properties for further off-chip analysis such as gene expression analysis.

### Microfluidics-based quantification of phenotypic and functional differences of 2D vs. 3D chemotaxis

**Fig 4:**
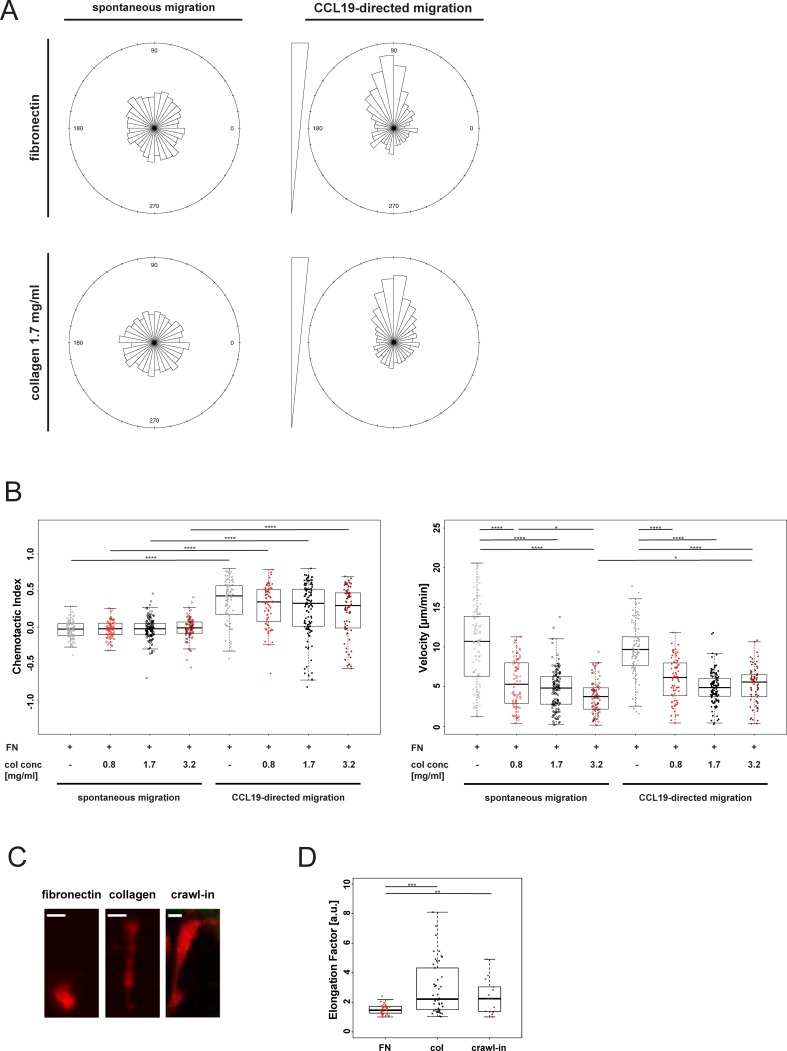
Quantification of microfluidic 2D and 3D migration. (A) Distribution of migration tracks of cells migrating on fibronectin (100 μg/mL) or in a collagen gel (1.7 mg/mL). Rose plot summarizing frequency of directions relative to the starting point. (B) Quantification of migration characteristics of cells migrating on fibronectin (100 μg/mL, FN) or in a collagen gel of various densities (col conc = collagen concentration). Chemotactic Index is a measure of chemotactic efficiency (calculated by dividing the distance from start to end point of cells in axis of the gradient by the total migration distance of every cell). Median and interquartile ranges shown, ρ values were calculated by Kruksal-Wallis test with Dunn’s multiple comparison test. Not significant (ns) ρ ≥ 0.05; * ρ < 0.05; **** ρ < 0.0001. (C) Representative images of DC morphology in microfluidic 2D and 3D environments and mouse ear tissue. Scale bar, 10 μm. (D) Quantification of cell morphology of cells migrating on fibronectin (100 μg/mL), in a collagen gel (1.7 mg/mL) or in mouse ear tissue. Elongation factor = cell length divided by its perpendicular cell width. FN = fibronectin, col = collagen 1.7 mg/mL, crawl-in = mouse ear crawl-in migration assay. Median and interquartile ranges shown, ρ values were calculated by Kruksal-Wallis test with Dunn’s multiple comparison test. ** ρ < 0.01; *** ρ < 0.001.

Cells migrating in 3D matrices are in contrast to cells that migrate on 2D surfaces not depending on the interaction of integrins with the pericellular environment [25]. Besides these biomechanical differences the pericellular microenvironment also dictates morphology and signal integration of migrating cells [8, 26]. These findings are based on assessing cells migrating spontaneously or in the presence of evenly distributed chemokines on 2D surfaces or in 3D collagen gels [25]. No information is available on differential migration properties of immune cells navigating in 3D compared to on 2D environments superimposed with similar chemokine gradients. We therefore aimed at functionally and phenotypically assessing migration of DCs in a collagen gel compared to migration on a 2D surface coated with fibronectin. As shown in **Fig 4A**, DCs migrated spontaneously non-directed in a microfluidic 2D and 3D environments. Exposure of DCs cultured on 2D or in 3D environments to gradients of the chemokine CCL19 induced in both conditions directed cell migration towards higher concentrations of the gradients (**S2 Movie**) as reflected by the directions of the migration trajectories (**Fig 4A**). This was accompanied in both, 2D and 3D environments with a comparable increase in directedness of cell migration when compared to spontaneously migrating cells (**Fig 4B**, panel 1). However, migration velocities were significantly decreased in DCs migrating within the collagen gels compared to cells migrating on a 2D surface (**Fig 4B**, panel 2). Next, we assessed migration properties of DCs loaded in different compartments of the migration chamber. We compared migration velocity and the chemotactic index of cells starting in the compartment closest to the chemokine source, in the middle compartment and in the compartment most distant from the chemokine source (sink). We assessed migration properties in cells migrating on a fibronectin-coated 2D surface and in collagen gels of differing concentrations for spontaneous migration and for migration in presence of a CCL19-gradient at 2 time intervals (30–50 min and 70–90 min). In absence of a chemokine gradient, cells migrated spontaneously non-directed in 2D and 3D environments in both time intervals (**S3A Fig**). Cell velocity was during spontaneous migration in a 2D environment increased compared to cells cultured in 3D environments in both time intervals (**S3A Fig**). Following exposure to a CCL19 gradient, cells in the source and the middle compartment of the migration chamber migrated towards higher concentrations of the chemokine as reflected by an increased chemotactic index in 2D and 3D environments in both time intervals (**S3B Fig**). The velocity of cell migration was comparable to cells migrating spontaneously with cells migrating on a 2D surface again faster than cells navigating in a 3D environment.

Analysis of the velocities of cells migrating in a titration of collagen gel density showed a dose-dependent effect of the collagen concentration on cell migration speed (**Fig 4B**, panel 1). These findings provide evidence that previously reported reduced spontaneous migration velocities of cells cultured in 3D when compared to 2D environments [27] apply also for cells navigating along chemokine gradients. This is also in line with data comparing the migration speed of DCs in a titration of collagen densities in a dish [8].

Phenotypically, cells migrating on a 2D surface had a rounded or ellipsoid morphology (**Fig 4C**; **S2 Movie**) that characterizes amoeboid cell migration [8]. In contrast to this, cells migrating in a 3D collagen matrix were characterized by the formation of long protrusions of up to 50 μm length (**Fig 4C**; **S2 and S3 Movies**). This resembles the morphology of cells migrating in murine tissues (**Fig 4C**). Quantification of cell morphologies revealed increased elongation ratios of cells migrating in our microfluidic 3D environment compared to cells migrating on a 2D surface (**Fig 4D**). These data correspond to findings in human monocytes migrating either on a 2D uncoated surface or in a collagen gel inside a u-slide chemotaxis chamber [28]. Importantly, the elongation ratios of DCs migrating in the microfluidic 3D environment were comparable to DCs migrating in murine tissue (**Fig 4D**). Quantification of the cell morphology of cells migrating in a titration of collagen gel density showed a dose-dependent effect of the collagen concentration on the elongation of migrating DCs (**S4 Fig**).

In summary, these findings provide evidence that migration characteristics of DCs migrating in microfluidic 3D environments are comparable to DCs migrating in murine tissues.

## Discussion

Three-dimensional cellular microenvironments are in comparison to classical 2D cell culture systems more representative of intact tissue matrices found in organisms [29, 30]. 3D-assays are of particular importance when studying biophysical processes in which the pericellular environment is known to influence cellular behaviour such as in cell migration. [31, 32]. Therefore, a number of cell culture systems have been developed to track cells migrating in 3D microenvironments [11]. Also, several microfluidic devices have been developed that allow exposure of cells cultured in 3D to soluble gradients [33–35]. However, spatiodeterministic retrieval of cells from these microfluidic chips was not possible in such setups.

In this study we addressed these limitations by developing a microfluidic device specifically engineered to study 3D migration of primary cells along precisely controllable chemokine gradients. The setup described here allows the polymerization of a 3D collagen gel containing dendritic cells in microfluidic migration chambers and subsequent generation of chemokine gradients along these collagen gels. By doing so we could show that cells migrating in a microfluidic collagen gel had a morphology that was comparable to cells migrating in animal tissue and is characterised by the formation of long protrusions. This morphology differed substantially from cells migrating on a 2D surface. The possibility to precisely control the generation and maintenance of chemokine gradients allowed us for the first time to compare in proof of principle experiments migration characteristics of DCs migrating on a 2D vs. in a 3D environment in similar gradients of the chemokine CCL19. Whether slower displacement of cells migrating in 3D along the chemokine gradient relates to differences of the substrates cells are migrating in or to physical hindrance of cells migrating in 3D remains to be determined. Against the background that microfluidics allow precise modification of surfaces cells are exposed to, our setup therefore qualifies for addressing basic biophysical aspects of cell migration related to the microenvironment. Such capabilities are important not only in studying immune cell migration but also when assessing mechanisms of tissue repair or cancer cell migration. Studying cell migration of adherent cancer cells by using microfluidics is well established [36–38] and can presumably also be assessed in our device, although we have so far not tested this. Taken together, our microfluidic 3D migration device has the potential to substantially contribute to a better understanding of the biophysical processes underlying locomotion of cells migrating in organ tissues.

## Supporting information

S1 Fig Collagen polymerization. (A) Collagen polymerization process over time visualized by anti-collagen antibody staining. Scale bar, 50 μm. (B) Fully polymerized collagen matrices of different concentrations, indicated conditions were stained with an anti-collagen antibody after polymerization. Scale bar, 25 μm. (C) Quantification of collagen staining shown in (B).
(EPS)

S2 Fig Sub-analysis reproducibility of microfluidic 3D migration assay. (A) Cells loaded in a collagen gel (1.7 mg/mL) were tracked for 90 min during diffusion-based soluble CCL19 gradient generation with maximal concentrations of 5 μg/mL. Included in this analysis are only cell initially positioned within the middle compartment (indicated in red). Migration tracks plotted to a common starting point for all migration chambers are shown. (B) Chemotactic Index as a measure of chemotactic efficiency and velocity are shown. Median and interquartile ranges shown, ρ values were calculated by Kruksal-Wallis test with Dunn’s multiple comparison test. Not significant (ns) ρ ≥ 0.05.
(EPS)

S3 Fig Analysis of cells in different chamber compartment migrating in 2D and 3D environments. (A) Migration characteristics of cells migration spontaneously on fibronectin (100 μg/mL, FN) or in a titration of collagen concentrations (col conc), cells are grouped according to their initial positon within the migration chamber during 2 time intervals (30–50 min and 70–90 min). Starting point source (green dots) are cells initially positioned in proximity to the chemokine gradient, middle (red dots) are cells placed in the middle compartment and sink (blue dots) are cells placed most far away from the chemokine source. Chemotactic Index as a measure of chemotactic efficiency and velocity are shown. (B) Identical analysis as shown in (A) for cells migrating in a CCL19 gradient (maximal concentration 5 μg/mL).
(EPS)

S4 Fig Cell morphology of cells migrating in CCL19 in different collagen gel densities. Quantification of cell morphology of cell migrating on fibronectin fibronectin (100 μg/mL, FN) or in a titration of collagen concentrations (col conc). Elongation factor = cell length divided by its perpendicular cell width. Median and interquartile ranges shown, ρ values were calculated by Kruksal-Wallis test with Dunn’s multiple comparison test. ** ρ < 0.01, *** ρ < 0.001.
(EPS)

S5 Fig Specificity of on-chip anti-MHC class II staining. Anti-MHC class II staining shown in parallel with bright field images on T cells (A), B cells (B), immature DCs (C) and mature DCs (D) loaded in collagen gel. Scale bar, 25 μm.
(EPS)

S1 Movie Collagen polymerization. Collagen polymerization process dynamically visualized by anti-collagen antibody staining. Scale bar, 50 μm.
(MOV)

S2 Movie CCL19-directed migration in microfluidic 2D and 3D environments. DCs migrating on fibronectin (100 μg/mL) (left panel) and in a collagen gel (1.7 mg/mL) (right panel) in a CCL19 gradient (maximal concentration 5 μg/mL). Migration tracks of individual cells are tracked for 120 min and indicated in different colors.
(MOV)

S3 Movie Cell morphology of cells migrating in CCL19 gradient in stained collagen. CellTracker Deep Red stained DCs migrating in stained collagen gel (1.7 mg/mL) in a CCL19 gradient (maximal concentration 5 μg/mL). Scale bar, 50 μm.
(MOV)
